# Bim and Bmf Synergize To Induce Apoptosis in *Neisseria Gonorrhoeae* Infection

**DOI:** 10.1371/journal.ppat.1000348

**Published:** 2009-03-20

**Authors:** Oliver Kepp, Kathleen Gottschalk, Yuri Churin, Krishnaraj Rajalingam, Volker Brinkmann, Nikolaus Machuy, Guido Kroemer, Thomas Rudel

**Affiliations:** 1 Department of Molecular Biology, Max Planck Institute for Infection Biology, Berlin, Germany; 2 Core Facility for Microscopy, Max Planck Institute for Infection Biology, Berlin, Germany; 3 INSERM, U848, Institute Gustave Roussy, Université Paris Sud, Paris, France; 4 Biozentrum, University of Würzburg, Department of Microbiology, Würzburg, Germany; Northwestern University Feinberg School of Medicine, United States of America

## Abstract

Bcl-2 family proteins including the pro-apoptotic BH3-only proteins are central regulators of apoptotic cell death. Here we show by a focused siRNA miniscreen that the synergistic action of the BH3-only proteins Bim and Bmf is required for apoptosis induced by infection with *Neisseria gonorrhoeae* (Ngo). While Bim and Bmf were associated with the cytoskeleton of healthy cells, they both were released upon Ngo infection. Loss of Bim and Bmf from the cytoskeleton fraction required the activation of Jun-N-terminal kinase-1 (JNK-1), which in turn depended on Rac-1. Depletion and inhibition of Rac-1, JNK-1, Bim, or Bmf prevented the activation of Bak and Bax and the subsequent activation of caspases. Apoptosis could be reconstituted in Bim-depleted and Bmf-depleted cells by additional silencing of antiapoptotic Mcl-1 and Bcl-X_L_, respectively. Our data indicate a synergistic role for both cytoskeletal-associated BH3-only proteins, Bim, and Bmf, in an apoptotic pathway leading to the clearance of Ngo-infected cells.

## Author Summary

A variety of physiological death signals, as well as pathological insults, trigger apoptosis, a genetically programmed form of cell death. Pathogens often induce host cell apoptosis to establish a successful infection. *Neisseria gonorrhoeae* (Ngo), the etiological agent of the sexually transmitted disease gonorrhoea, is a highly adapted obligate human-specific pathogen and has been shown to induce apoptosis in infected cells. Here we unveil the molecular mechanisms leading to apoptosis of infected cells. We show that Ngo-mediated apoptosis requires a special subset of proapoptotic proteins from the group of BH3-only proteins. BH3-only proteins act as stress sensors to translate toxic environmental signals to the initiation of apoptosis. In a siRNA-based miniscreen, we found Bim and Bmf, BH3-only proteins associated with the cytoskeleton, necessary to induce host cell apoptosis upon infection. Bim and Bmf inactivated different inhibitors of apoptosis and thereby induced cell death in response to infection. Our data unveil a novel pathway of infection-induced apoptosis that enhances our understanding of the mechanism by which BH3-only proteins control apoptotic cell death.

## Introduction

Infection with various pathogens results in the inhibition or activation of apoptotic cell death [1]. Whereas viral pathogens frequently inhibit host cell apoptosis, many bacteria kill immune or epithelial cells by apoptosis allowing them to subvert immune reactions or to invade tissues, respectively. The obligate human specific bacterium *Neisseria gonorrhoeae* (Ngo), the causative agent of the sexually transmitted disease gonorrhea, induces apoptosis in genital epithelia. Since induction of apoptosis requires the firm attachment of the gonococci to host cells [2], exfoliation of infected epithelial cells covered with adherent bacteria has been suggested as the immediate cellular responses against infection [3],[4]. This detachment-associated apoptosis of infected cells resembles anoikis, a special form of apoptosis that is induced by absent or inappropriate cell–matrix interactions [5].

Bcl-2 family proteins control mitochondrial outer membrane permeabilization (MOMP), which is the critical step in many forms of apoptosis [6],[7]. The Bcl-2 family consists of pro- and antiapoptotic members that share homologies within their Bcl-2 homology domains (BH). The antiapoptotic Bcl-2 family proteins harbor BH1-4 domains and presumably act by sequestering and inhibiting proapoptotic Bcl-2 members [8]. Proapoptotic Bcl-2 family proteins can be further subdivided into the branch of pore forming, multidomain BH1-3 proteins (like Bak and Bax) and the BH3-only branch (including Bim, Bmf, Bid, Bad, Noxa and Puma) [9],[10]. Active BH3-only proteins cause conformational changes within Bak and Bax, which subsequently homooligomerize and form pores in the outer mitochondrial membrane [11],[12]. MOMP culminates in the release of proapoptotic proteins like cytochrome *c*, leading to the activation of caspases and caspase-independent death effectors [13].

The mechanisms through which BH3-only proteins activate Bak or Bax are not fully understood. BH3-only proteins may release Bak and Bax from inhibition by anti-apoptotic Bcl-2 protein [14]. Alternatively, the group of BH3-only proteins may include two subgroups, namely survival antagonists that neutralize BH1-4 proteins, and death agonists that activate BH1-3 proteins [15],[16]. A competition of death agonists and survival antagonists for the binding to BH1-4 proteins has been reported [17]. Upon binding of survival antagonists, death agonists are released from their sequestration by BH1-4 proteins and hence freed to act directly on BH1-3 proteins.

Cytotoxic stimuli activate BH3-only proteins by a variety of distinct mechanisms such as p53-dependent transcriptional regulation (Puma and Noxa [18],[19]), proteolytic cleavage (Bid [20]), dephosphorylation (BAD [21]) or phosphorylation (Bim and Bmf). Under normal circumstances, Bim and Bmf are sequestered via dynein light chains (DLC) to the actin and tubulin cytoskeleton, respectively, which prevents them from activating Bak and Bax [22],[23]. Previous work has suggested that phosphorylation of Bim and Bmf within their DLC-binding sites is mediated by Jun-N-terminal kinase-1 (JNK-1) facilitating the release of both proteins from the cytoskeleton during anoikis [24],[25].

Here, we analyzed the signaling pathways upstream of Bak and Bax in Ngo-infected cells. Unexpectedly, both Bim and Bmf were found to act in concert to induce apoptosis of Ngo infected cells. Our data suggest a role of Ngo infection-induced cytoskeletal reorganization in the initiation of apoptosis pathways.

## Results

### Caspase-independent detachment of infected cells

**Figure 1:**
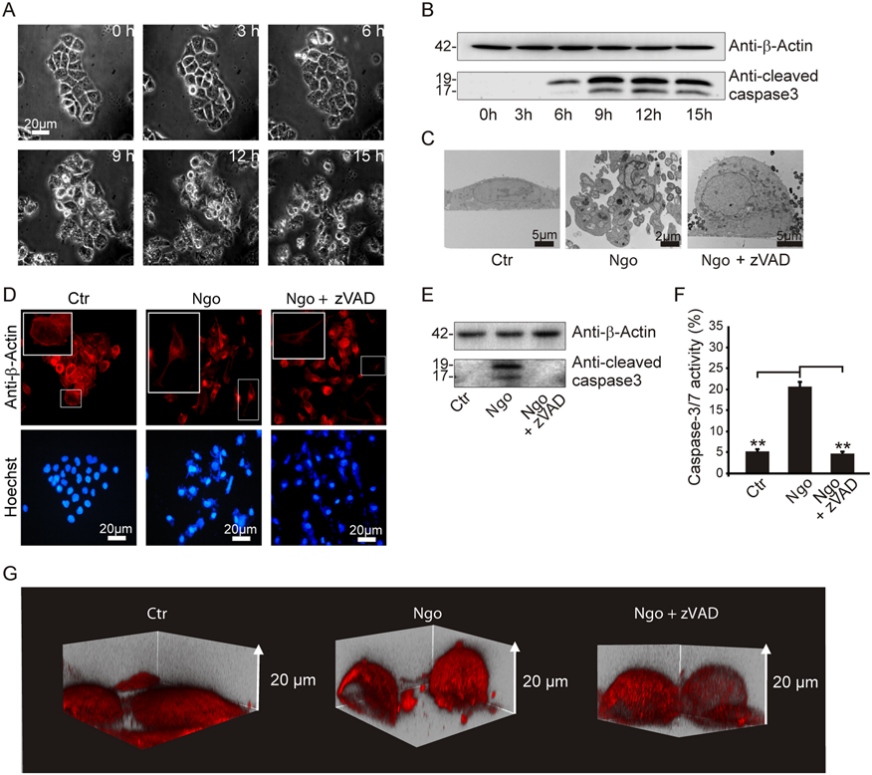
Caspase-independent loss of matrix attachment. (A) Phase contrast pictures of one HeLa colony during the time course of an infection with Ngo shows detachment upon 6 h of treatment and apoptotic morphologies upon 9–12 h post-infection. (B,E,F) Caspase activation during infection and effective caspase inhibition by Z-VAD-fmk was shown by immunoblot detection of cleaved caspase-3 and FACS analysis using CaspACE. The FACS data represent the mean±SD of at least three independent experiments. (C) Electron micrographs depicting matrix loss were taken 15 h post-infection. Z-VAD-fmk pretreatment was used to exclude caspases-dependent effects. (D,G) Cytoskeletal changes were visualized by immunofluorescence microscopy using an actin-specific antibody. 3D remodeling of respective confocal z stacks was performed using Imaris software.

Exfoliation of epithelial cells has previously been described to be caused by Ngo infection [3],[4],[26]. Since this process resembles anoikis, we further investigated the connection between Ngo-induced cell detachment and apoptosis. HeLa cells were infected with Ngo VPI (N242), a clinical isolate and morphological changes were correlated with the activation of caspases. Detachment from the culture support was visible as soon as 6 to 9 h post-infection (Figure 1A) concomitant with the proteolytic maturation of caspase 3 (Figure 1B). To test whether caspase activity is required for exfoliation, cells were infected in the absence or presence of the pan-caspase inhibitor Z-VAD-fmk and then were analyzed by electron and fluorescence microscopy. In the presence of Z-VAD-fmk, Ngo-infected cells continued to detach yet remained otherwise intact and hence failed to disintegrate by apoptosis (Figure 1C and 1D, Video S1) while the activation of caspases 3 and 7 was blocked during the entire duration of the experiment (Figure 1E and 1F). Detachment was further analyzed by acquiring z stacks of infected cells by laser scanning confocal microscopy and subsequent 3-dimensional remodeling (Figure 1G and Video S2). The detachment and induction of apoptosis is not a general response of these cells to infection stress since the same cell line exhibits marked apoptosis resistance as consequence of infection with *C. trachomatis* [27],[28]. These results demonstrate that caspases are required for the apoptotic disassembly of Ngo-infected cells but not for their detachment.

To test whether the observed effect is specific for HeLa cells and the bacterial strain VPI (N242), we tested other gonococcal derivatives for their capacity to induce exfoliation and cell death. Eleven clinical gonococcal isolates from different patients isolated from blood, urethra, cervix, vagina or urine were analyzed. The capacity of these strains to adhere to HeLa cells correlated well with the induction of exfoliation and apoptosis (Table 1; Figure S1), suggesting that exfoliation and apoptosis induction is a common effect of adherence to HeLa cells. We also investigated whether the effect is specific for HeLa cells. N242 induced significant apoptosis in ME180 and Hep2 cells. From the genetically defined derivatives of the laboratory strain MS11, only strain N920 which forms pili induced apoptosis in ME180 cells, whereas strains expressing Opa57 (N1163, not shown) or Opa52 (N309) failed to induce significant apoptosis in HeLa, Hep2 and ME180 cells (Figure S2). N242 is a clinical isolate expressing 5 different Opacity-associated (Opa) proteins required for the binding of these bacteria to heparane sulfate proteoglycanes (HSPG), integrins or ‘carcinoembryonic antigen-related cell adhesion molecules’ (CEACAMs) of the host cell ([29]; for review see [30]). We next tested whether laboratory strains gain the capacity to induce detachment of HeLa cells expressing Opa protein receptors. The MS11 strain MS11 N927 (Opa^−^; PorB_IA_) failed to induce detachment of HeLa cells permanently expressing CEACAM 1 or CEACAM 3 respectively (Figure S3) [31]. In contrast, the MS11 strain N1163 (Opa57; PorB_IA_) induced detachment of infected cells in the CEACAM 1 expressing HeLa cells but not in the cell line expressing CEACAM 3 (Figure S3). These data suggested an interaction-, and in addition a receptor-specific mechanism underlying detachment and apoptosis induction.

**Table 1:** Induction of exfoliation and caspase-3 activity by clinical isolates.

Strain Number	Isolate	Adherence^a^	Detachment^b^	Caspase-3^c^
VP1 (N242)	n.d.	+	+	+
MZ155/04	Blood	+	+	+
MZ359/05	Urethra	+	+	+
MZ441/05	Urethra	−	−	−
MZ308/06	Cervix	+	+	+
MZ452/06	Vagina	−	−	−
MZ489/06	n.d.	+	+	+
MZ552/06	n.d.	+	+	+
MZ38/07	Urethra	−	−	−
MZ114/07	Urine	++	+	+
MZ245/07	n.d.	+	+	+

a Adherence was determined by plating assays of Saponine lysed infected cells.

b Detachment was determined by microscopical assays.

c Infection induced p17/p19 active caspase-3 fragments and PARP cleavage determined by immunoblotting

+, adherence similar as VP1; ++, adherence at least 10-fold more efficient as VP1; −, no significant adherence; n.d., not documented.

### Synergistic action of BH3-only proteins during apoptosis

**Figure 2:**
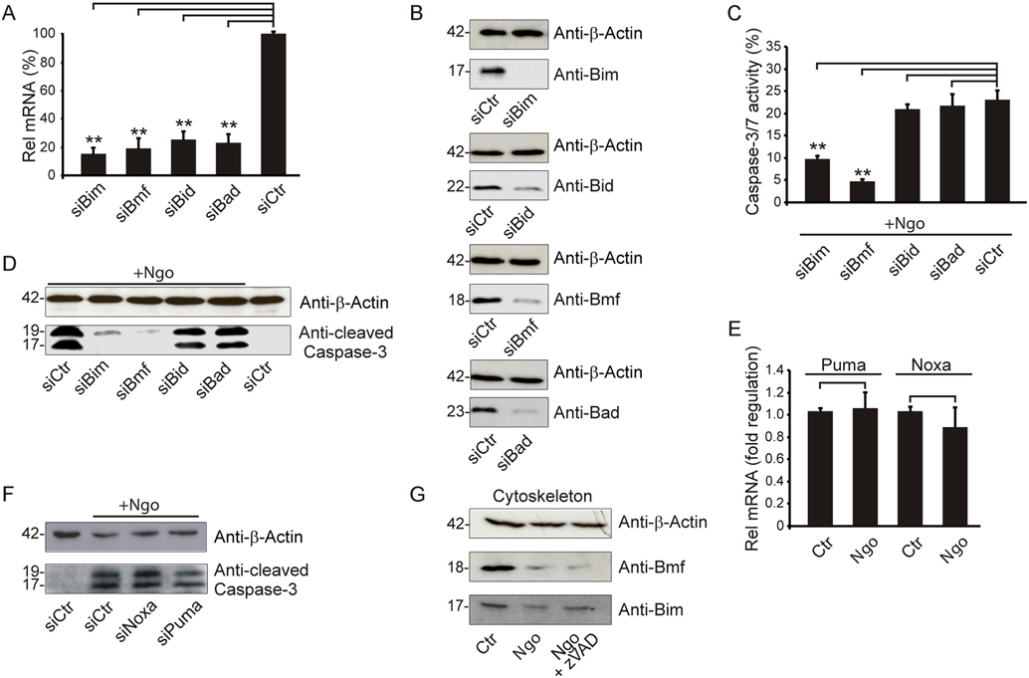
Bim and Bmf mediate Ngo-induced apoptosis. (A,B) HeLa cells were treated with siRNA, and 72 h post-transfection RNA was prepared and the knockdown efficiency was validated by qRT-PCR. The data represent the mean±SD of three independent experiments. Whole cell lysates were prepared for immunoblot detection of the indicated antigens. (C,D) siRNA-transfected cells were infected with Ngo (N242) for 15 h with an multiplicity of infection (MOI) of 1. The caspase activity was measured by FACS analysis using the CaspACE assay. Data represent the mean±SD of at least three independent experiments. Whole cell lysates were taken at the same time point post-infection and the activity of caspase 3 was analyzed using an anti-cleaved caspase 3 antibody. Equal loading was ensured using the indicated loading control. (E) Puma and Noxa transcriptional regulation was examined by qRT-PCR 15 h post-infection (Ngo) versus non-infected cells (Ctr). Data represent the mean±SD of three independent experiments. (F) Expression of Noxa (siNoxa) and Puma (siPuma) was silenced by RNA interference, and the caspase activity was monitored in infected cells. (G) The cytoskeletal fraction of infected and zVAD pretreated cells were checked together with untreated controls. Bim, Bmf, and the indicated loading control were detected using specific antibodies.

Since infection of HeLa cells with N242 caused the most prominent effects, we focused on this system to further investigate the mechanisms underlying infection-induced apoptosis. We have previously demonstrated that infection with Neisseria induced the activation of Bak and Bax and finally apoptotic cell death [32]. To delineate the signaling pathway leading to the activation of Bak and Bax, we systematically depleted BH3-only proteins in a RNA interference miniscreen. The knockdown of the siRNAs was validated by quantitative real-time PCR (>75% knockdown at the mRNA level) and immunoblot analysis (>75% knockdown at the protein level) (Figure 2A and 2B). Knockdown of Bim and Bmf (but not that of Bid or Bad) resulted in a significant reduction of effector caspase activity, as measured with a fluorogenic caspase 3/7 substrate or by immunochemical detection of proteolytically mature caspase 3 (Figure 2C and 2D, and Figure S4). Bim and Bmf knockdown specifically inhibited the caspase activation induced by Ngo (Figure 2C and 2D, and Figure S5), yet had no effect on caspase activation induced by the genotoxic agent cisplatin (Figure S5A). Ngo infection failed to induce Puma, Noxa and any of the tested mRNAs for BH3 only proteins (Figure 2E, and Figure S6), although cisplatin was able to activate the transcription of both the Puma and Noxa genes (Figure S5B and Figure S5C). Accordingly, the depletion of Puma or Noxa did not affect caspase-3 activation in Ngo-infected cells (Figure 2F). These data demonstrate that Bim and Bmf are specifically required for the Ngo-triggered activation of caspases.

### Release of Bim and Bmf from the cytoskeleton during infection

In healthy cells, Bim and Bmf are sequestered to the cytoskeleton by binding to dynein light chains. In response to cytotoxic stimuli, that induce cytoskeletal rearrangements, Bim and Bmf may act as stress sensors in thus far that they are released from the cytoskeleton and induce the activation of Bak and Bax [22],[23]. Accordingly, cytoskeleton fractions obtained from Ngo-infected cells generally contained less Bim and Bmf than those from non-infected control cells (Figure 2G and Figure S7A). A similar result was obtained when cytoskeleton and cytosol were separated by sucrose gradient centrifugations. Bim and Bmf from infected samples shifted from heavier cytoskeleton containing to lighter fractions (Figure S7B), indicating a release of these proteins from the cytoskeleton in infected cells. Addition of Z-VAD-fmk did not prevent the release of Bim and Bmf from the cytoskeleton (Figure 2G), demonstrating that this phenomenon occurs independently of caspase activity.

### JNK-dependent activation of Bim and Bmf

**Figure 3:**
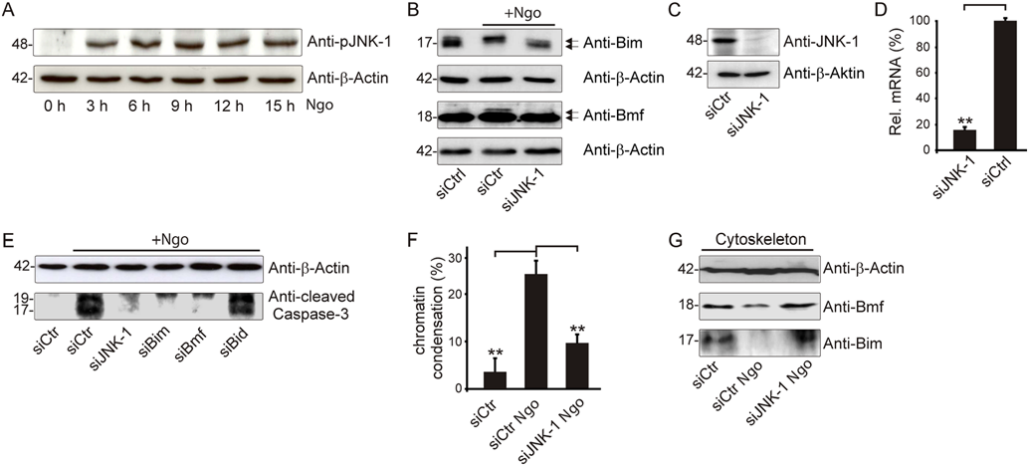
JNK mediates Bim and Bmf activity. (A) JNK phosphorylation and post-translational modification of Bim at the indicated time points during the course of infection were visualized using the indicated antibodies. (B) Modification of Bim and Bmf 15 h post-infection with and without JNK was studied by SDS PAGE and Western blot analysis with the respective antibodies. Arrows indicate the two detected forms of Bim and Bmf. (C,D) The JNK-dependent modification of Bim and Bmf was analyzed using siRNA-mediated knockdown validated by Western blot and qRT-PCR. (E) An effect of JNK knockdown on caspase activation upon infection was shown by the use of an antibody specific for the large subunit of mature caspase-3. SiRNAs against Bim, Bmf, and Bid were used as positive and negative controls, respectively. (F) The effect of JNK-1-knockdown on apoptosis induction was analyzed upon siRNA treatment. The fraction of cells showing condensed/fragmented chromatin was counted in five microscopical fields and three independent experiments using Hoechst 33342. (G) Cytoskeletal extracts were analyzed for Bim and Bmf in JNK knockdowns, infected, and uninfected controls using the indicated antibodies.

Active JNK-1 is reportedly sufficient for the release of Bim and Bmf from the cytoskeleton [24]. We have previously shown that JNK-1 is activated already 30 minutes after infection with *Neisseria*, leading to NFκB activation and proinflammatory responses [33]. Although the short-term effects of JNK-1 activation can be cytoprotective, prolonged JNK activation induces apoptotic cell death [34]. Phosphorylated, active JNK-1 could be detected for the whole period of Ngo infection up to 15 h (Figure 3A), correlating with a reduced electrophoretic mobility of Bim and Bmf at later timepoints (Figure 3B and Figure S8). ERK seems not to be involved in the signaling as there was no activation upon infection (Figure S9). Silencing of JNK-1 with validated siRNAs (Figure 3C) prevented the shift in the size of Bim and Bmf (Figure 3B, 3C, and 3D), indicating a role of JNK-1 in post-translational modification of Bim-L and Bmf in Ngo-infected cells.

The caspase activity of Ngo-infected cells depleted of JNK was reduced to the same level as that of cells subjected to the knockdown of Bim or Bmf (Figure 3E). Moreover, the frequency of cells with apoptotic chromatin condensation was reduced In JNK-1-depleted as compared to control cells (Figure 3F). JNK-1 depletion also partially inhibited the Ngo-induced release of Bim and Bmf from the cytoskeleton (Figure 3G). In addition inhibiting JNK-1 by means of a chemical inhibitor partially reduced a translocation of Bim and Bmf from heavier to lighter fractions in sucrose gradients (Figure S7C). In conclusion, JNK-1 depletion can prevent the post-transcriptional modification of Bim and Bmf, reduce their loss from the cytoskeleton fraction and inhibit apoptosis of Ngo-infected cells.

### Rac required for cytoskeletal and apoptotic signaling

**Figure 4:**
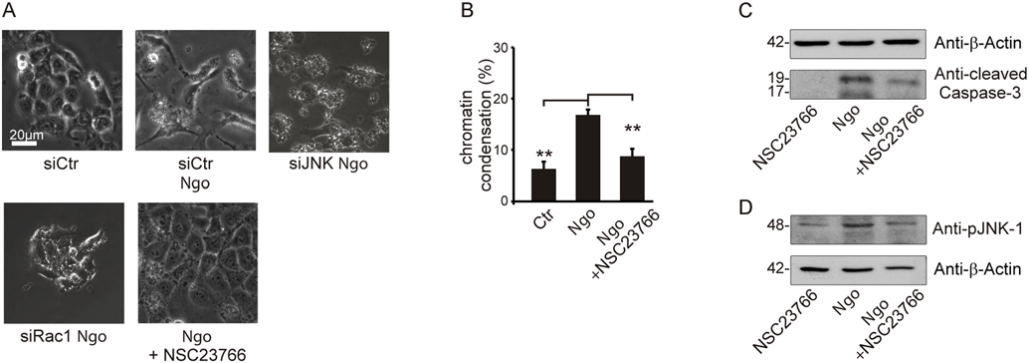
Rac-1 required for cytoskeletal changes and apoptotic signaling. (A) Shown are phase contrast pictures depicting the respective cytoskeletal phenotype of untreated control cells, cells transfected with siJNK-1, siRac1, and treated with the Rac inhibitor NSC23766 15 h post-infection. (B) Inhibition of apoptosis in infected, NSC23766 pretreated cells was quantified by determining the ratio of cells with condensed and normal chromatin by fluorescent microscopy. (C,D) The effect of Rac inhibitor NSC23766 on JNK activation and subsequent caspase cleavage was determined by Western blot using the indicated antibodies.

The Rho-GTPases are central regulators of cytoskeletal changes initiated by extracellular signals. Most prominent, Rho and Rac have been shown to be involved in neisserial uptake and phagocytosis [35]. Therefore, we reanalyzed the link between Rac and JNK signaling during Ngo-induced apoptosis [36]. The knockdown of Rac-1 or its inhibition with the pharmacological agent NSC23766 abolished the reorganization of the cytoskeleton initiated by Ngo infection (Figure 4A). Rac inhibition caused a significant reduction in caspase activation, apoptotic nuclear fragmentation (Figure 4B and 4D), JNK-1 phosphorylation (Figure 4D) and Bim and Bmf cytoskeletal release (Figure S7D). These experiment place Rac upstream of JNK-1 and all JNK-1-dependent apoptotic events affecting Ngo-infected cells.

### Bim and Bmf triggered activation of Bak and Bax

**Figure 5:**
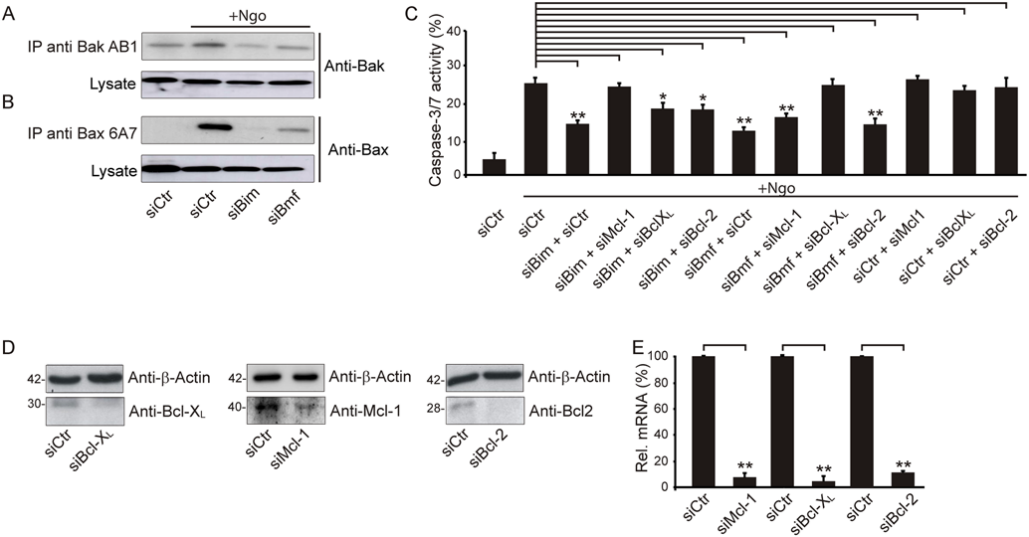
Bim-specific and Bmf-specific targeting of Mcl-1 and Bcl-X_L_. (A,B) The activation of Bak and Bax upon siRNA-mediated knockdown was visualized 15 h post-infection by immunoprecipitation with conformation-specific antibodies followed by SDS-PAGE and immunodetection with the indicated antibodies. (C) The network of Bcl-2 family proteins was analyzed by caspase activation assays after single or double knockdowns, 15 h post-infection. Shown are the means±SD of three independent experiments. (D,E) Knockdowns were validated by Western blot using the indicated antibodies (D) and qRT-PCR (E).

The role of Bim and Bmf in the activation of Bak and Bax – direct activation or deinhibition? - is still a matter of controversy [8],[15],[17]. Irrespective of their exact of mode of action the function of Bim and Bmf in Ngo-induced apoptosis cannot be redundant because depletion of either of them prevented the induction of apoptosis by Ngo infection. To unravel the mechanisms of this non-redundancy, we assessed the activation of Bak and Bax by means of antibodies that recognize their exposed N-termini and hence their activated conformation. SiRNA- and shRNA-mediated knockdown of Bim as well as siRNA-mediated knockdown of Bmf prior to Ngo infection prevented the activation of both Bak and Bax (Figure 5A and 5B and Figure S4), underlining the essential need of both Bim and Bmf in this pathway.

In certain apoptosis pathways like Ngo infection or cisplatin induction, Bak and Bax become activated in a hierarchical manner, with Bak acting upstream of Bax [32],[37]. The activation of Bak involves its release from antiapoptoticBcl-2 analogues such as Mcl-1 [38]. Combined silencing of Mcl-1 and Bim, but not that of Mcl-1 and Bmf or Mcl-1 knockdown alone reestablished the apoptotic program triggered by Ngo infection (Figure 5C), suggesting that Bim acts as a specific Mcl-1 antagonist in this system. Combined silencing of Bcl-X_L_ plus Bmf, but not that of Bcl-X_L_ and Bim or Bcl-X_L_ alone also reestablished Ngo-induced apoptosis (Figure 5C). In contrast, Bcl-2 co-silencing had no apoptosis-sensitizing effect on either Bim- or Bmf-depleted cells. Potential off target effects within the same protein family could be excluded by systematic cross analysis. In particular, Bim- and Bmf silencing did not cause deregulated expression of anti-apoptotic members of the Bcl-2 family (Figure S10), ruling out an indirect effect of Bim and Bmf depletion by overexpression of apoptosis inhibitors. We concluded from these data that Bim and Bmf activate apoptotic pathways by functionally sequestering Mcl-1 and Bcl-X_L_, respectively.

## Discussion

*Neisseria gonorrhoeae* is a highly adapted human pathogen that utilizes multiple adhesins to interact with host cell receptors to trigger cytoskeletal reorganization, invasion or phagocytic uptake, intraphagosomal accommodation, nuclear reprogramming of host cells, cytokine/chemokine release and finally host cell apoptosis [39]. By investigating the apoptotic pathway involved in the infection-induced activation of Bak and Bax, we discovered an unexpected connection between pathogen-induced cytoskeletal reorganization and apoptosis. Attachment of bacteria initiated the activation of Rac-1 leading to rearrangement of the cytoskeleton (which is presumably required for exfoliation) and the activating phosphorylation of the stress kinase JNK-1. JNK-1 then participated in the activation of the BH3-only proteins Bim and Bmf that together facilitate Bak- and Bax-dependent apoptosis.

Besides the well characterized isolate N242 [29], several other clinical isolates induced exfoliation and apoptosis indicating that gonococci trigger similar pathways leading to cell death. Our preliminary data on the initial trigger of cell detachment leading to cell death unveiled a role of specific adhesin – receptor interactions. N242 induced exfoliation and cell death in different cell lines tested. These effects very likely depend on the interaction of one or more of the expressed Opa proteins with a yet uncharacterized receptor. Although derivatives of strain MS11 failed to induce apoptosis in HeLa cells, a similar efficient response as with N242 was observed with derivative N1163 (Opa57; PorB_IA_) upon infection of HeLa-CEACAM1 but not in HeLa-CEACAM3. Interestingly, CEACAM-1 has been shown to be upregulated in primary ovarian surface epithelial cells by gonococcal infection suggesting that the interaction with this receptor has in vivo relevance [40]. Moreover, the specificity for one CEACAM-recombinant cell line over the other is interesting, because both have been demonstrated to be susceptible for infection with Opa57-expressing gonococci [41]. It is therefore likely that particular adhesin-receptor interactions determine the detachment and apoptosis induction as consequence of this cell – pathogen interaction. This assumption would be in agreement with several reports on the inhibition of apoptosis by gonococcal infection [42]. In one of these studies, Bim was downregulated upon infection of epithelial cells with a piliated gonococcal derivative [43], supporting a central role of Bim in life-death decisions as consequence of gonococcal infections.

Numerous bacterial pathogens induce the reorganization of the host cell cytoskeleton, often initiating the active uptake of bacteria [44]. Nevertheless, the activation of apoptosis is not a common outcome of such bacterial infections. Our data suggest that downstream of cytoskeletal reorganization, the prolonged activation of JNK-1 is required for lethal signaling. It is interesting to note that short term activation of JNK induces antiapoptotic and proinflammatory responses in the host cell infected with Ngo [33],[45]. JNK may therefore exert a dual function during Ngo infection, first by protecting the cell for a short period post-infection and then by triggering the exfoliation of the infected cells.

We show here that JNK was required for Bim- and Bmf-dependent apoptosis during infection, consistent with the previously described JNK-specific phosphorylation of Bim and Bmf within their dynein binding domains [24]. Accordingly, the release of Bim and Bmf from the cytoskeleton as well as their reduced electrophoretic mobility was reduced in JNK-1-depleted cells (Figure 3G).

**Figure 6:**
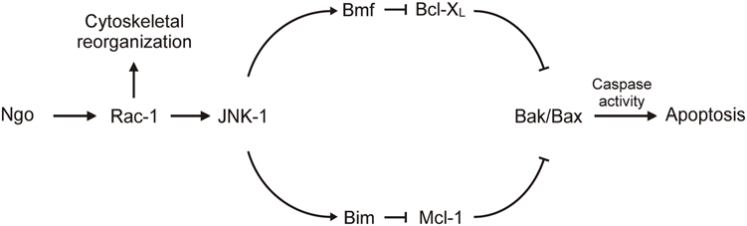
Model of Bim-dependent and Bmf-dependent apoptosis during Ngo-induced apoptosis. Ngo infection leads to a Rac-dependent activation of JNK-1 and a concurrent alteration of the cytoskeletal morphology. Upon JNK-1–mediated phosphorylation, the cytoskeleton-attached proapoptotic proteins Bim and Bmf are released. Subsequently, the antiapoptotic effects of Mcl-1 and Bcl-X_L_ are abrogated by Bim and Bmf, respectively, leading to the activation of Bak and Bax and cell death.

The exact mode of BH3-only activity is still being discussed. Here we show that both Bim and Bmf are essential to induce Bak and Bax activity for Ngo-triggered apoptosis. As the double knockdown of Bim and Mcl-1 re-sensitized cells for apoptosis, the action of Bmf alone seems to trigger apoptosis efficiently in the absence of Mcl-1. Likewise, apoptosis could be rescued in the absence of Bmf by co-knockdown of Bcl-X_L_, suggesting that the action of Bim alone suffices to induced apoptosis in the absence of Bcl-X_L_. In this scenario, both Bim and Bmf need to be activated for efficient induction of cell death due to their joint capacity to inhibit two different anti-apoptotic Bcl-2 homologues (see model in Figure 6). As a result, this study furnishes yet another example for the complex relationship between antagonizing pro- and antiapoptotic Bcl-2 family proteins.

## Materials and Methods

### Cell culture, bacterial strains, and infection

HeLa cells (human cervix carcinoma, later diagnosed as adenocarcinoma) ATCC CCL2 and HeLa cell lines expressing recombinant CEACAM receptors [31] were grown in RPMI 1640 (Gibco) supplemented with 10% heat inactivated FCS in the presence of 5% CO_2_. The cells were routinely passaged every 2–3 days and the passage number never exceeded 20 passages before a new batch with a low passage number was used. Cells were seeded 24 h before infection and were washed several times with RPMI before infection. HEK 293T cells (immortalized human embryonic kidney) ATCC CRL-11268 for the production of virus and HEp-2 (ATCC CCL-23) were grown in DMEM (Gibco) supplemented with 10% heat inactivated FCS. and ME-180 (HTB-33) were grown in McCoys 5a supplemented with 10% heat inactivated FCS respectively under the same conditions.

The following *N. gonorrhoeae* strains and derivatives were used in this study: The clinical isolate Ngo strain VP1 (N242; PorB_IA_; P^−^; Opa_27_; Opa_27,5_;Opa_28_; Opa_29_; Opa_30_; LPS type L1) [29]; Ngo strain MS11 derivatives N302 (PorB_IB_; P^−^; Opa^−^), N920 (PorB_IA_; P^+^; Opa^−^), a PorB_IA_ derivative of N917 (PorB_IB_; P^+^; Opa^−^) and N927 (PorB_IA_; P^−^; Opa^−^) have been described [46],[47]. MS11 derivative N1163 (PorB_IA_; P^−^; Opa_57_) is a PorB_IA_ derivative of strain N313 [41]. Clinical gonococcal isolates from Germany were obtained anonymously from the strain collection of the National Reference Laboratory for Meningococci hosted by the Institute for Hygiene and Microbiology at the University of Würzburg. Species confirmation for those strains was obtained at the Reference Laboratory by standard biochemical tests and partial 16S rRNA sequencing. Gonococci were grown on GC agar base plates (Becton Dickinson, Difco and Remel) supplemented with Proteose Pepton Nr. 3 (Difco) and 1% vitamin mix for 14–20 h at 37°C in 5% CO_2_ in a humidified atmosphere. Infections were routinely performed in the absence of FCS at a multiplicity of infection (MOI) of 1. If not indicated else wise the respective assays were carried out after 15 h of infection with N242.

For the inhibition of caspases, cells were pre-incubated with 50 µM Z-VAD-fmk (Bachem) for 15 min prior to infection and throughout the experiment. Cisplatin was used at a concentration of 50 µM for 20 h in supplemented media.

### Western blot

5×10^5^ cells per sample were harvested in 100 µl loading buffer (60 mM Tris-HCl pH 8.0, 6% SDS, 10 mM DTT, 6% β-mercaptoethanol, 40% glycerol, and 0.1% bromophenol blue) and 20 µl of the protein lysates were separated and transferred as described before [32]. The following antibodies and sera were used in this study: anti-β-Actin (Sigma); anti-Bad (Cell Signaling); anti-Bak NT (Upstate); anti-Bak (Ab-1) (Millipore);anti-Bax NT (Upstate); anti-Bax (6A7) (BD Pharmingen); anti-Bid (Cell Signaling); anti-Bim (Sigma); anti-Bmf (Cell Signaling); anti-cleaved Caspase-3 (Cell Signalling); anti-JNK-1 (Santa Cruz); anti-pJNK-1 (Cell Signalling) and anti-Mcl-1 (BD Pharmingen). Equal loading was routinely confirmed by appropriate loading controls.

### FACS assays

Caspase 3 and 7 activities were measured by CaspACE assay. Control and infected cells were collected and stained with 10 µM CaspACE (Promega) in growth media at 37°C, 5% CO_2_ for 20 min. After staining, cells were washed twice with PBS and immediately subjected to FACS analysis.

### Immunoprecipitation

Immunoprecipitation was carried out as described earlier [32]. Solubilized cells were precleared and incubated with 2 µg of anti-Bax (6A7) or anti-Bak (Ab1) antibody at 4°C for 2 h. Immunoprecipitates were collected by incubating with protein G-Sepharose (Amersham) for 2 h. The pellets were washed intensively with lysis buffer and resuspended in sample buffer before analysis by Western blotting using anti-Bax NT and anti-Bak NT antibodies as described above.

### RNA interference

5×10^5^ HeLa cells were transfected with 1 µg of siRNA (Quiagen) using RNAiFect transfection kit (Quiagen) according to the manufacturer's instructions. The gene silencing was routinely validated by real time PCR as previously described [48] and by Western blot analysis 72 h post-transfection. The sequence targeted by siBad; siBim, siBid; siBmf; siJNK; siNoxa; siPuma; siMcl-1; siBcl-X_L_, Bcl-2 and siRac-1 were: ACGAGTTTGTGGACTCCTTTA; CGGAGACGAGTTTAACGCTTA; TAGGGACTATCTATCTTAATA; CACCGGCTTCATGTGCAGCA; AAGAAGCUAAGCCGACCAUUU; TGGGCTATATACAGTCCTCAA; CAGCCTGTAAGATACTGTATA; CGGGACTGGCTAGTTAAACAA; TCCATTATAAGCTGTCGCAGA; ATGCATTTCCTGGAGAATATA and GAGCTTTGAACAGGTAGTGAA respectively.

### Generation of stable shRNA-expressing HeLa

Stable shRNA-expressing HeLa cells were generated as described before in Kepp et al., 2007. The DNA coding for the shRNA was cloned into pLVTH-M vectors from which it integrated upon viral transfer into the genome of target cells. GFP was used as a marker to select for stable clones. The following sequence was targeted to silence Bim by shRNA expression: TAAGATAACCATTCGTGGG. The efficiency of gene silencing was validated by Western blot analysis and quantitative realtime PCR. The cells transduced with the empty vector were used as controls.

### Immunofluorescence microscopy

Cells seeded on coverslips were infected for 15 h, fixed in 3.7% PFA and permeabilized using 0.1% Triton X-100. Nonspecific binding was blocked by using 1% goat serum. The samples were stained using anti-actin (Sigma) and anti-tubulin (molecular probes) followed by detection with fluorochrome-coupled secondary antibodies (Jackson Immuno Research) using a Leica confocal microscope with TCS software or a Zeiss immunofluorescence microscope with ACT software. 3dimensional remodeling was performed using Metamorph and Imaris software.

For apoptosis quantification, fixed cells were stained with 1 µg/ml Hoechst 33342 (Invitrogen) for 10 min followed by intense washing with PBS. A minimum of 5 fields per slide was analyzed for chromatin condensation using a Zeiss immunofluorescence microscope.

### Transmission electron microscopy

Control, infected and infected zVAD treated cells were fixed 15 h post-infection with 2.5% glutaraldehyde, post-fixed with 0.5% osmium tetroxide and contrasted using tannic acid and uranyl acetate. Specimens were dehydrated in a graded ethanol series and embedded in Polybed. Ultrathin sections were analyzed in a Leo 906E transmission electron microscope (Leo GmbH).

### Cytoskeletal preparation

To analyze cytoskeleton-associated proteins 1×10^6^ cells were incubated for 15 min in HBSS (Gibco). All lipidic membranes were destabilized for 5 min at 4°C by incubation with 5 ml high detergent containing Buffer M (1 mM EGTA, 4% PEG 6000, 100 mM PIPES pH 6.9) containing 0.5% Triton X-100. The cytoplasmic and compartmental proteins containing supernatant was removed and the cytoskeleton was washed with cold Buffer M. The remaining proteins were collected in sample buffer and analyzed by Western blotting.

### Statistical analysis

The averages and standard errors of the mean as well as the t-tests have been calculated using MS Excel. Significance is indicated with ** p < 0,01 and * p < 0,05. If not indicated else wise in the figure legend the data represents at least 3 independent experiments.

## Supporting Information

Figure S1 Activation of caspase 3 by clinical gonococcal isolates. Isolates MZ522/06; MZ155/04; MZ441/05; MZ489/06; MZ359/05; MZ38/07; MZ114/07; MZ308/06; MZ245/07; and MZ452/06 were used to infect HeLa cells for 15 h and active caspase 3, and cleaved caspase 3 substrate PARP was detected by immunoblotting (see Materials and Methods for details). Infection with N242 and N242 in the presence of the caspase inhibitor zVAD (N242 + zVAD) was used as positive and negative control, respectively. Actin was detected on the same blots as loading control.
(0.39 MB TIF)

Figure S2 N242-induced apoptosis in epithelia cells. HeLa, Me180, and Hep2 cells were infected with the indicated neisserial strains and the induction of apoptosis was analyzed by quantification of fragmented chromatin using Hoechst staining and fluorescence microscopy. The neisserial strain N242 caused significant apoptosis in all analyzed cell lines, whereas other strains failed to cause significant apoptosis in all cell lines.
(0.16 MB TIF)

Figure S3 Specific Opa-receptor interaction required for induction of cell detachment and apoptosis. (A) Fluorescence-activated cell sorting (FACS) analysis of CEACAM expression by HeLa-CEACAM1 and HeLa-CEACAM3. Control (HeLa) and recombinant cells expressing CEACAM1 (CEA1) or CEACAM3 (CEA3) were detached with accutase and incubated with mouse anti-ceacam antibody (clone D14HD11) and anti-mouse Cy2 (red line). Isotope controls (black lines) were only incubated with anti-mouse-Cy2. (B) Induction of apoptosis depends on specific CEACAM-Opa interaction. Recombinant HeLa cell lines were either left uninfected (Ctr) or infected with N927 (Opa-;P-) or N1163 (Opa57;P-). Opa57 expressing bacteria adhered to both cell lines (not shown) as previously published by Billker et al., 2002. Apoptotic cells with condensed chromatin were quantified by microscopy. Shown is one typical example of several experiments with similar results. (C) Infected recombinant cell lines were analyzed for cell detachment by phase contrast microscopy.
(0.68 MB TIF)

Figure S4 Bim is necessary for Ngo-induced apoptosis. (A) Stable shRNA-expressing HeLa cells were generated by viral transfer and subsequent clonal selection. The knockdown was validated by qRT-PCR. The data represent the mean±SD of three independent experiments. (B) Depletion of Bim prevented the activation of Bak and Bax as well as subsequent Caspase-activation upon Ngo infection. shBim and control cells harboring an empty vector were infected for 15 h, and the activity of Bak and Bax was analyzed by immunoprecipitation using conformation-specific antibodies. Caspase activity was shown by immunodetection of the cleaved forms of caspases 3.
(0.13 MB TIF)

Figure S5 DNA damage induced BH3-only signaling. (A) siRNA transfected cells were treated with 20 µM cisplatin for 20 h, and the caspase activity was measured by FACS using CaspACE assay. The data represent the mean±SD of at least three independent experiments. (B) Puma and Noxa mRNA levels were examined by qRT-PCR and showed significant upregulation 20 h post-treatment. The data represent the mean±SD of three independent experiments. (C) Protein levels were determined by Western blot with the indicated antibodies, demonstrating an increased protein level of both proteins 20 h after cisplatin treatment.
(0.27 MB TIF)

Figure S6 Neisserial infection causes no transcriptional regulation of BH3-only proteins. The effect of infection on the mRNA levels of the BH3-only proteins was analyzed by qRT-PCR and showed no significant changes in comparison with uninfected controls.
(0.06 MB TIF)

Figure S7 Release of Bim and Bmf from their sequestration sites. (A) F-actin-enriched P1 and myosin-enriched P2 fractions were separated from 107 cells as described by Puthalakath et al. (Science 293: 1829–1832, 2001). The fractions were analyzed by Western blot, with the indicated antibodies, showing less Bmf and less Bim in P1 upon infection, whereas the level of Bim in P2 seems to stay unchanged. (B) Gradient centrifugation was carried out as described in Puthalakath et al., 2001. The 4 ml gradient was divided into 0.5 ml fractions, which were then analyzed by Western blot with the indicated antibodies. Bim and Bmf translocate upon infection from heavier fractions to lighter fractions of the gradient. (C) The inhibition of JNK using the chemical JNK-1 Inhibitor-1 (Calbiochem) at 1 µM during the experiment also inhibited the translocation of Bim and Bmf to lighter fractions of the gradient. (D) Same as under (C) using the Rac-1 inhibitor NSC23766.
(0.99 MB TIF)

Figure S8 Electrophoretic mobility of Bim changes at late time points. HeLa cells were infected, and samples were taken after the indicated time. The electrophoretic mobility of Bim was analyzed by Western blot, showing Bim phosphorylation no earlier than 9 h post-infection.
(0.09 MB TIF)

Figure S9 Exclusion of ERK-specific effects. ERK activation was analyzed in untreated and Ngo-infected samples in the presence and in the absence of the ERK-specific Inhibitor UO126. Western blot analysis showed no significant activation of ERK by infection with Ngo.
(0.07 MB TIF)

Figure S10 Exclusion of off-target effects and cross-regulation. HeLa cells were transfected with siBim and siBmf. The knockdown of the specific gene products as well as potential off-target effects and cross-regulation within the Bcl-2 protein family were analyzed by Western blot with antibodies detecting the indicated proteins. No cross-regulation or off-target effects of the siRNA treatment could be detected.
(0.16 MB TIF)

Video S1 Infection-induced changes in cellular shape. Actin-GFP (Clonetech)-expressing cells were infected and a series of z-stacks was acquired every hour using an Olympus spinning disc microscope. The pictures were 3-dimensional remodeled using the Imaris software. Shown is one representative cell undergoing morphological changes.
(1.9 MB AVI)

Video S2 Cytoskeletal changes prior to cell death. Cells were cultured in glass-bottom dishes and placed under an Olympus spinning disc microscope. Phase contrast pictures of the cells were taken every 10 min upon infection.
(2.3 MB AVI)
