# Supplementary figures and images for: Bim and Bmf Synergize To Induce Apoptosis in Neisseria Gonorrhoeae Infection
Source: PLoS Pathog. 2009 Mar 20;5(3):e1000348. doi: 10.1371/journal.ppat.1000348 (PMC2654407; doi:10.1371/journal.ppat.1000348)

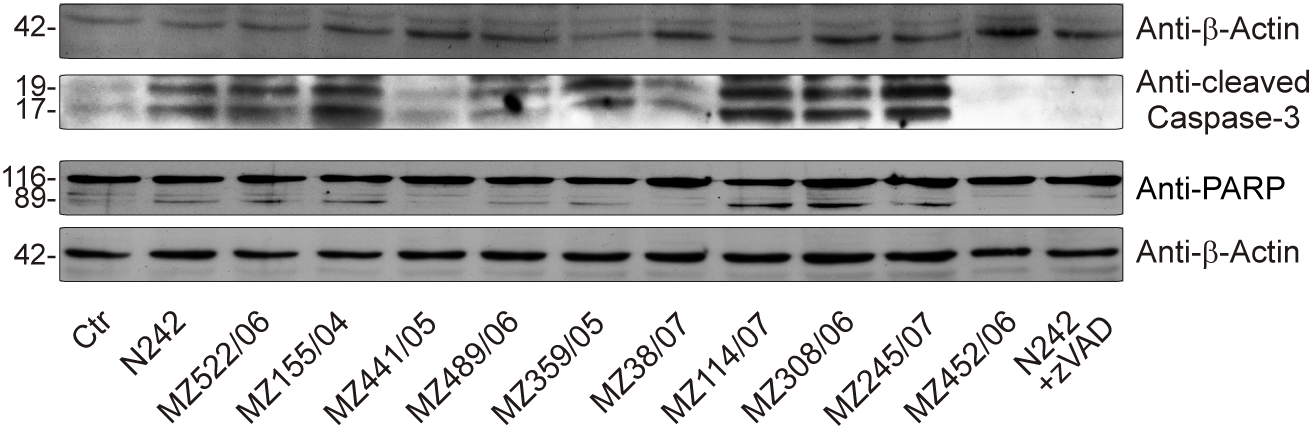

Supplement: Figure S1 — Activation of caspase 3 by clinical gonococcal isolates. Isolates MZ522/06; MZ155/04; MZ441/05; MZ489/06; MZ359/05; MZ38/07; MZ114/07; MZ308/06; MZ245/07; and MZ452/06 were used to infect HeLa cells for 15 h and active caspase 3, and cleaved caspase 3 substrate PARP was detected by immunoblotting (see Materials and Methods for details). Infection with N242 and N242 in the presence of the caspase inhibitor zVAD (N242 + zVAD) was used as positive and negative control, respectively. Actin was detected on the same blots as loading control. (0.39 MB TIF) [file ppat.1000348.s001.tif]

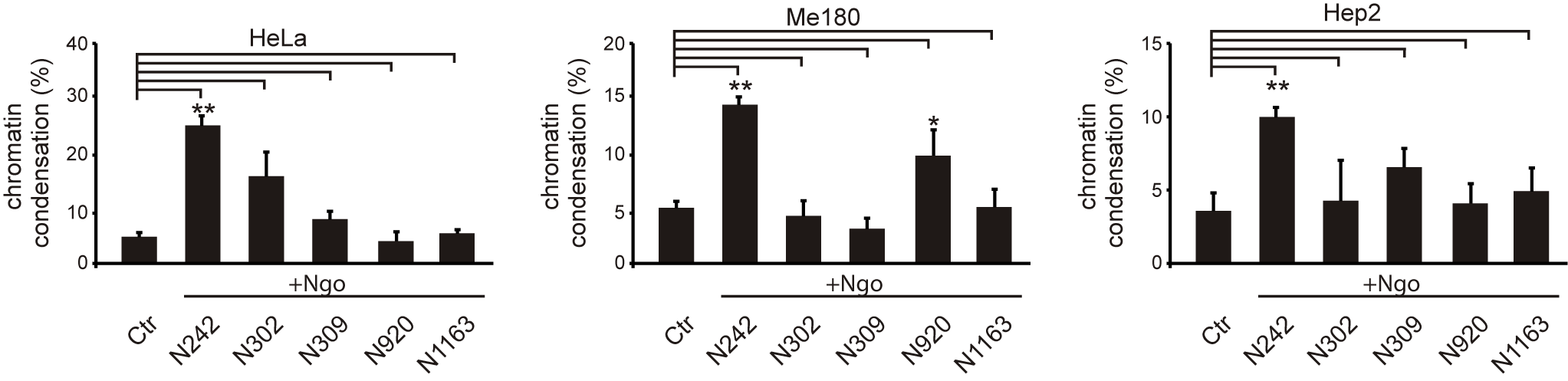

Supplement: Figure S2 — N242-induced apoptosis in epithelia cells. HeLa, Me180, and Hep2 cells were infected with the indicated neisserial strains and the induction of apoptosis was analyzed by quantification of fragmented chromatin using Hoechst staining and fluorescence microscopy. The neisserial strain N242 caused significant apoptosis in all analyzed cell lines, whereas other strains failed to cause significant apoptosis in all cell lines. (0.16 MB TIF) [file ppat.1000348.s002.tif]

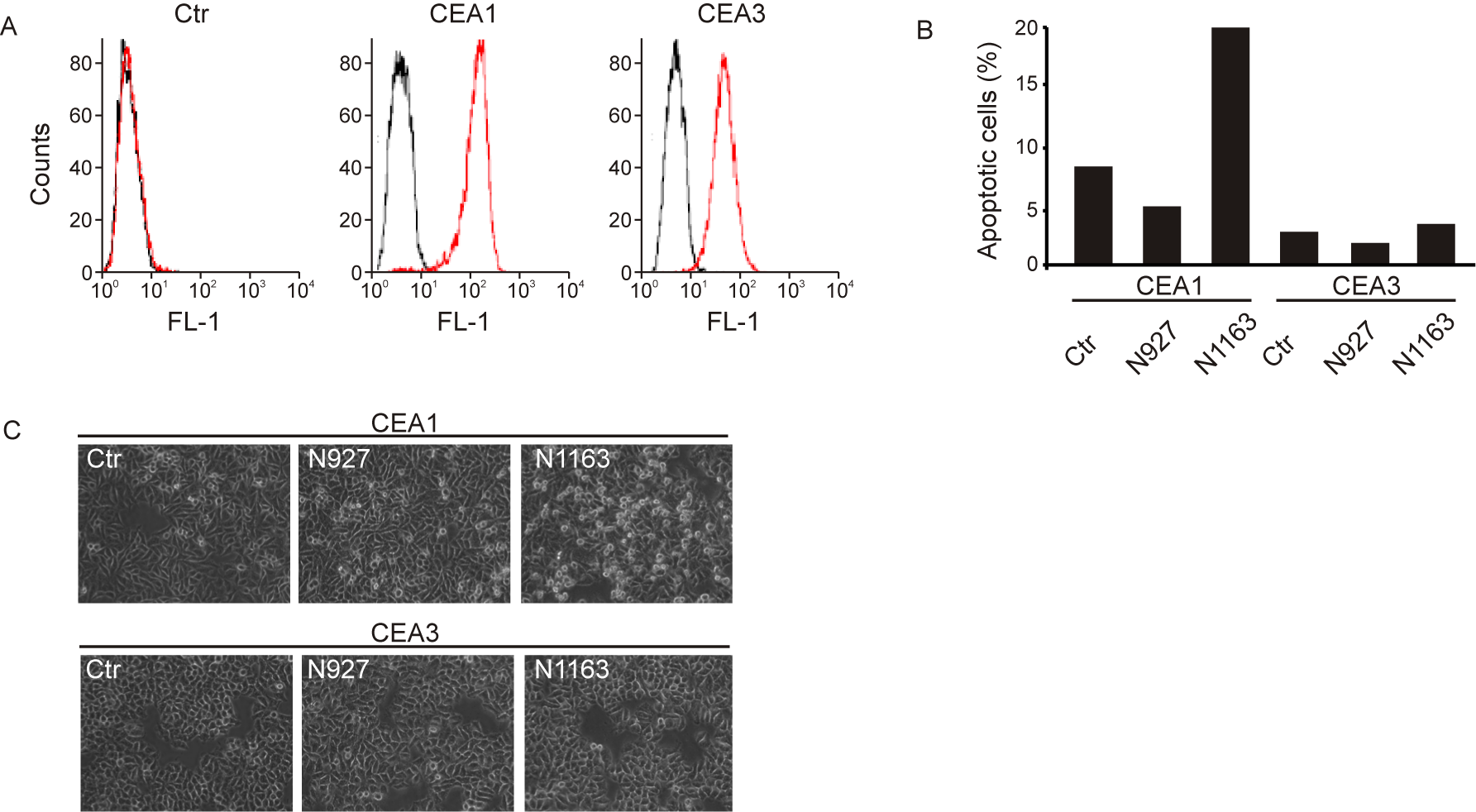

Supplement: Figure S3 — Specific Opa-receptor interaction required for induction of cell detachment and apoptosis. (A) Fluorescence-activated cell sorting (FACS) analysis of CEACAM expression by HeLa-CEACAM1 and HeLa-CEACAM3. Control (HeLa) and recombinant cells expressing CEACAM1 (CEA1) or CEACAM3 (CEA3) were detached with accutase and incubated with mouse anti-ceacam antibody (clone D14HD11) and anti-mouse Cy2 (red line). Isotope controls (black lines) were only incubated with anti-mouse-Cy2. (B) Induction of apoptosis depends on specific CEACAM-Opa interaction. Recombinant HeLa cell lines were either left uninfected (Ctr) or infected with N927 (Opa-;P-) or N1163 (Opa57;P-). Opa57 expressing bacteria adhered to both cell lines (not shown) as previously published by Billker et al., 2002. Apoptotic cells with condensed chromatin were quantified by microscopy. Shown is one typical example of several experiments with similar results. (C) Infected recombinant cell lines were analyzed for cell detachment by phase contrast microscopy. (0.68 MB TIF) [file ppat.1000348.s003.tif]

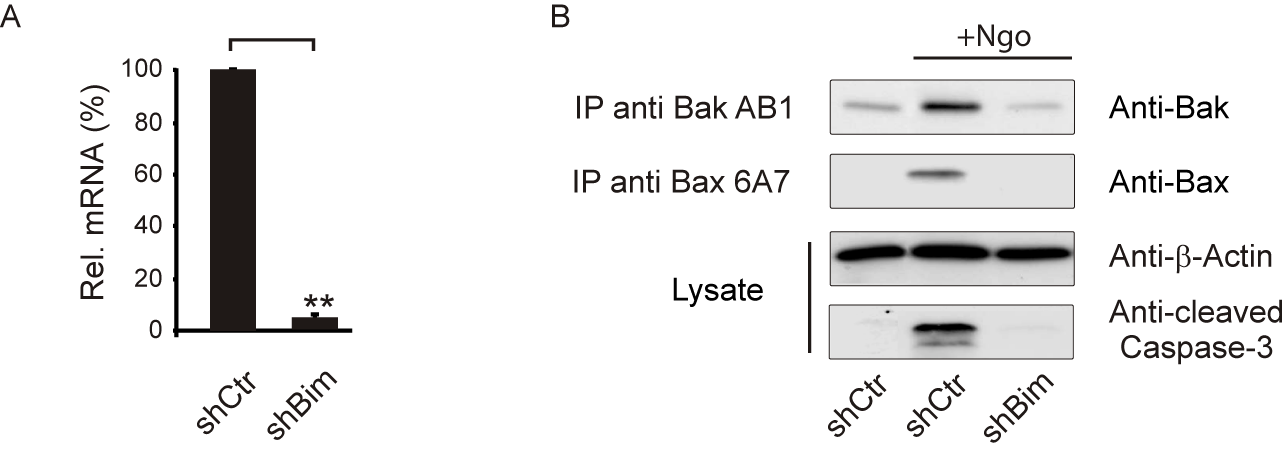

Supplement: Figure S4 — Bim is necessary for Ngo-induced apoptosis. (A) Stable shRNA-expressing HeLa cells were generated by viral transfer and subsequent clonal selection. The knockdown was validated by qRT-PCR. The data represent the mean±SD of three independent experiments. (B) Depletion of Bim prevented the activation of Bak and Bax as well as subsequent Caspase-activation upon Ngo infection. shBim and control cells harboring an empty vector were infected for 15 h, and the activity of Bak and Bax was analyzed by immunoprecipitation using conformation-specific antibodies. Caspase activity was shown by immunodetection of the cleaved forms of caspases 3. (0.13 MB TIF) [file ppat.1000348.s004.tif]

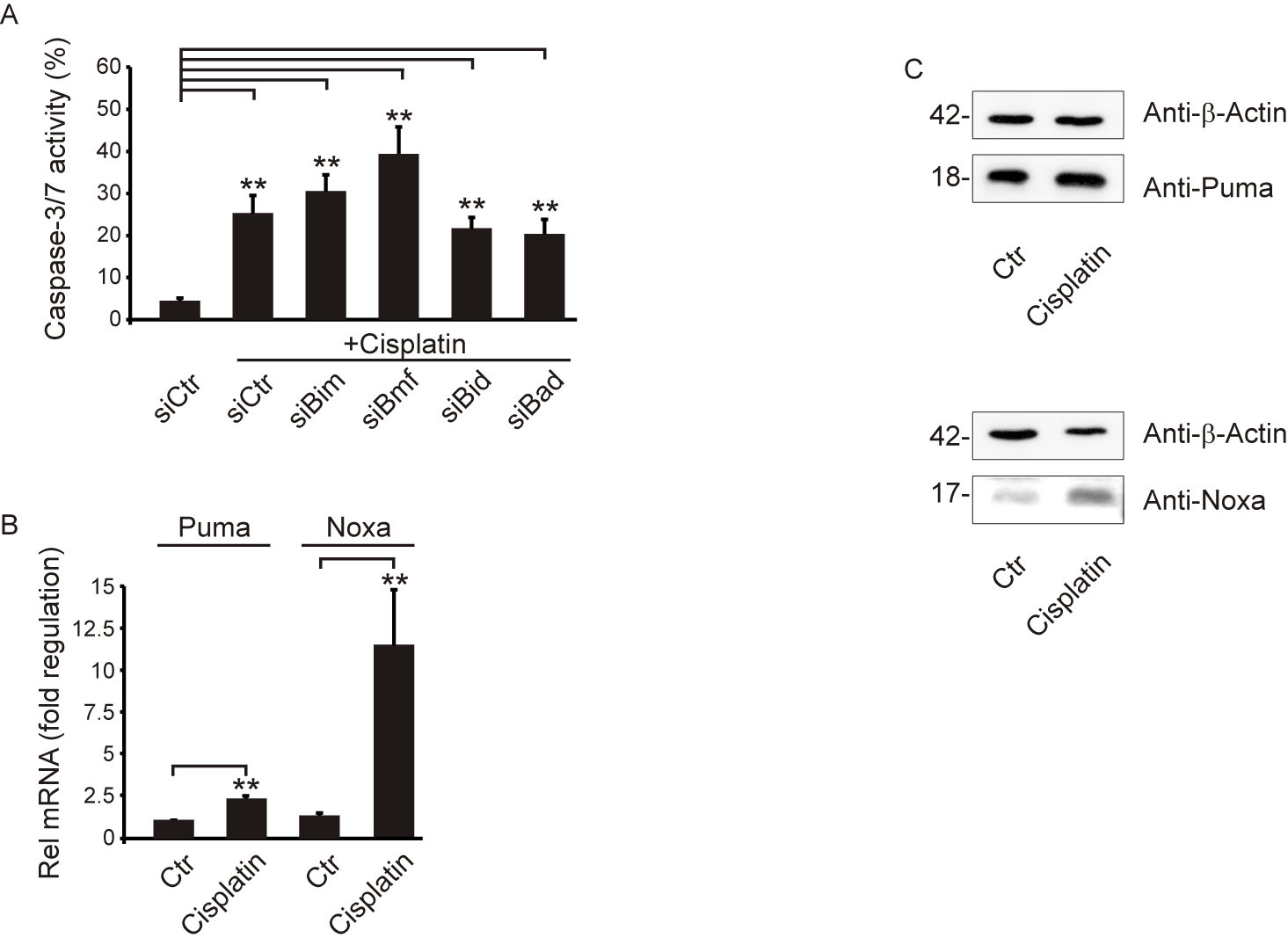

Supplement: Figure S5 — DNA damage induced BH3-only signaling. (A) siRNA transfected cells were treated with 20 µM cisplatin for 20 h, and the caspase activity was measured by FACS using CaspACE assay. The data represent the mean±SD of at least three independent experiments. (B) Puma and Noxa mRNA levels were examined by qRT-PCR and showed significant upregulation 20 h post-treatment. The data represent the mean±SD of three independent experiments. (C) Protein levels were determined by Western blot with the indicated antibodies, demonstrating an increased protein level of both proteins 20 h after cisplatin treatment. (0.27 MB TIF) [file ppat.1000348.s005.tif]

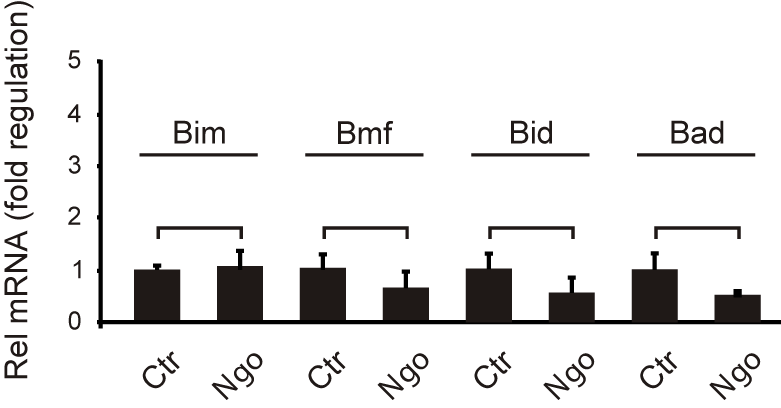

Supplement: Figure S6 — Neisserial infection causes no transcriptional regulation of BH3-only proteins. The effect of infection on the mRNA levels of the BH3-only proteins was analyzed by qRT-PCR and showed no significant changes in comparison with uninfected controls. (0.06 MB TIF) [file ppat.1000348.s006.tif]

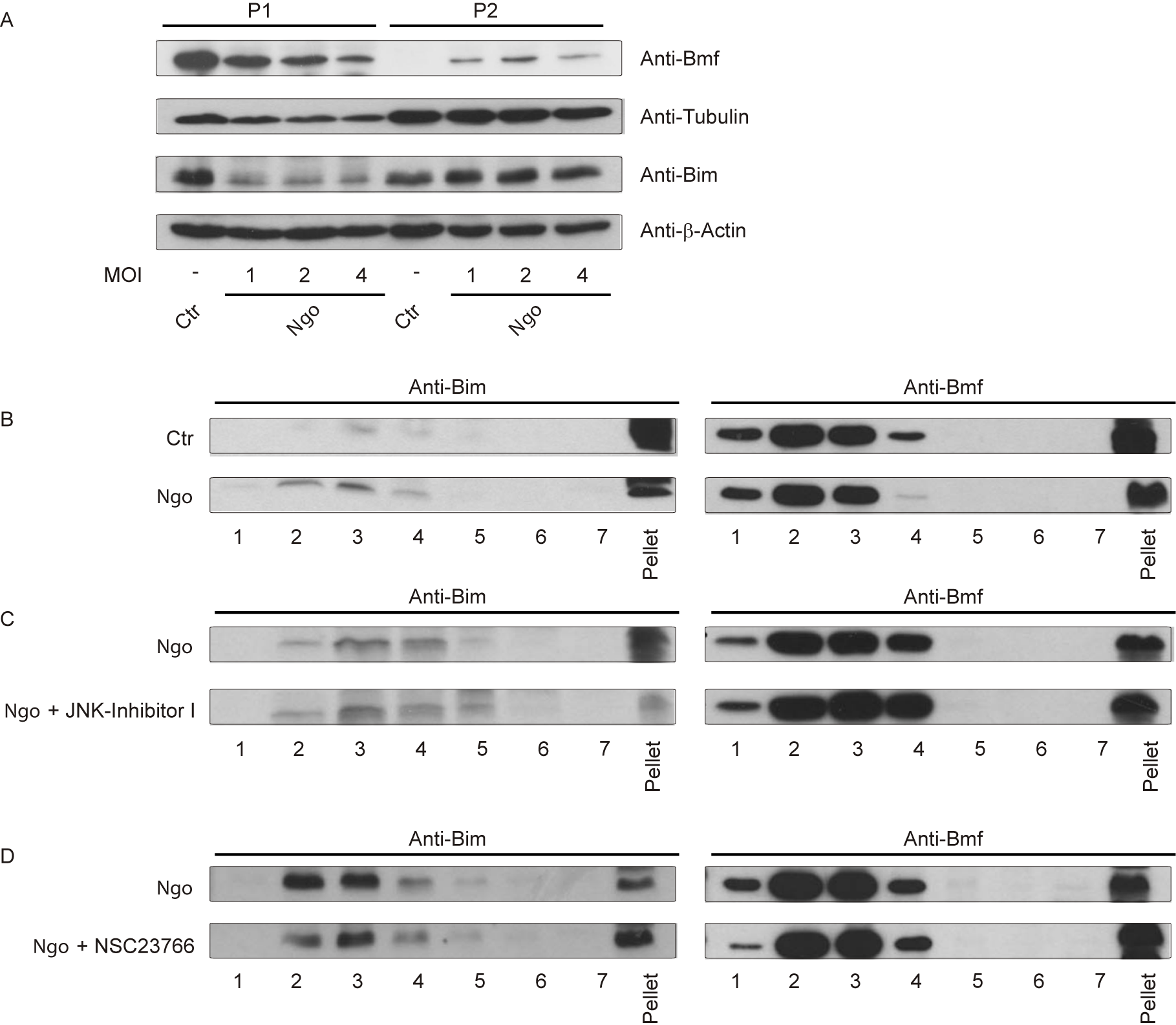

Supplement: Figure S7 — Release of Bim and Bmf from their sequestration sites. (A) F-actin-enriched P1 and myosin-enriched P2 fractions were separated from 107 cells as described by Puthalakath et al. (Science 293: 1829–1832, 2001). The fractions were analyzed by Western blot, with the indicated antibodies, showing less Bmf and less Bim in P1 upon infection, whereas the level of Bim in P2 seems to stay unchanged. (B) Gradient centrifugation was carried out as described in Puthalakath et al., 2001. The 4 ml gradient was divided into 0.5 ml fractions, which were then analyzed by Western blot with the indicated antibodies. Bim and Bmf translocate upon infection from heavier fractions to lighter fractions of the gradient. (C) The inhibition of JNK using the chemical JNK-1 Inhibitor-1 (Calbiochem) at 1 µM during the experiment also inhibited the translocation of Bim and Bmf to lighter fractions of the gradient. (D) Same as under (C) using the Rac-1 inhibitor NSC23766. (0.99 MB TIF) [file ppat.1000348.s007.tif]

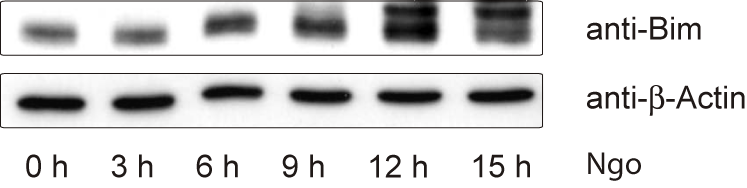

Supplement: Figure S8 — Electrophoretic mobility of Bim changes at late time points. HeLa cells were infected, and samples were taken after the indicated time. The electrophoretic mobility of Bim was analyzed by Western blot, showing Bim phosphorylation no earlier than 9 h post-infection. (0.09 MB TIF) [file ppat.1000348.s008.tif]

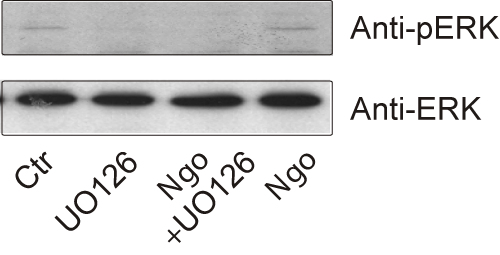

Supplement: Figure S9 — Exclusion of ERK-specific effects. ERK activation was analyzed in untreated and Ngo-infected samples in the presence and in the absence of the ERK-specific Inhibitor UO126. Western blot analysis showed no significant activation of ERK by infection with Ngo. (0.07 MB TIF) [file ppat.1000348.s009.tif]

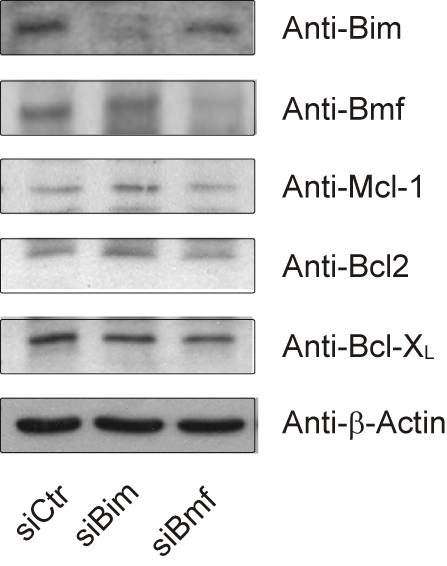

Supplement: Figure S10 — Exclusion of off-target effects and cross-regulation. HeLa cells were transfected with siBim and siBmf. The knockdown of the specific gene products as well as potential off-target effects and cross-regulation within the Bcl-2 protein family were analyzed by Western blot with antibodies detecting the indicated proteins. No cross-regulation or off-target effects of the siRNA treatment could be detected. (0.16 MB TIF) [file ppat.1000348.s010.tif]
